# Slowly growing solitary neurofibroma of the thumb: A case report

**DOI:** 10.1097/MD.0000000000023611

**Published:** 2021-01-15

**Authors:** Kwang Seog Kim, Dong Gyu Lee, Do Hun Lee, Jae Ha Hwang, Sam Yong Lee

**Affiliations:** Institution: Department of Plastic and Reconstructive Surgery, Chonnam National University Medical School, Gwangju, Republic of Korea.

**Keywords:** hand, nerve sheath tumors, neurofibroma

## Abstract

**Rationale:**

Neurofibromas can develop as part of neurofibromatosis or as a solitary tumor. Although solitary neurofibromas generally grow slowly, they rarely grow for more than 10 years, and such tumors have not been described in the hand.

**Patient concerns:**

A 60-year-old woman presented with a mass on the dorsum of the proximal phalanx of the right thumb that had been enlarging since more than a decade.

**Diagnoses:**

Preoperative imaging revealed a moderately defined soft tissue mass, which measured 1.5 cm × 1.5 cm × 0.7 cm, with no bone and joint abnormalities on the right thumb. The final diagnosis of the tumor was solitary neurofibroma without evidence of neurofibromatosis.

**Intervention:**

*En bloc* resection of the tumor was performed through a longitudinal skin incision.

**Outcomes:**

After surgery, the patient had no complaints of pain but had a temporary tingling sensation. After 14 months of follow-up, no signs of recurrence of the tumor were observed and she was highly satisfied with the results of the surgery.

**Lessons:**

Solitary neurofibroma is quite rare, especially one in the hand. However, in dealing with soft tissue tumors of the hand, particularly those with neurologic symptoms, neurofibroma should be included in the differential diagnosis.

## 1 Introduction

Although neurofibromas consist of hamartomatous proliferations of neuro-mesenchymal components, including Schwann cells, fibroblasts, perineural cells, and mastocytes, the proportion of the cell types varies between cases.^[1]^ First described by Von Recklinghausen, neurofibromas can occur as solitary or multiple tumors, and may be associated with neurofibromatosis.^[2]^ Solitary neurofibromas typically develop asymptomatically as slowly-enlarging soft growths in the second or third decade of life.^[3]^ However, a solitary neurofibroma slowly growing over a decade is extremely rare, and such a tumor has not been described in the hand. This paper reports a case of neurofibroma that grew slowly along the dorsal digital branch of the radial nerve of the thumb.

## 2 Case presentation

**Figure 1:**
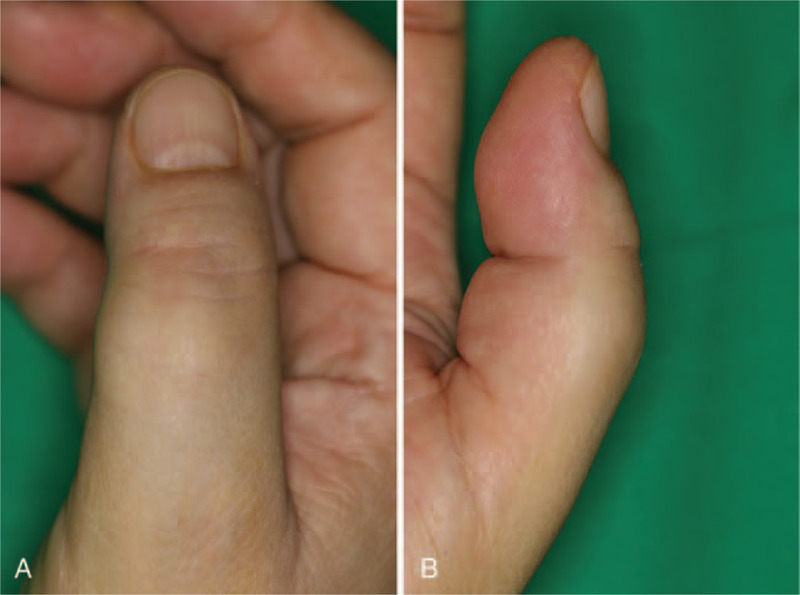
Preoperative photographs showing a mass on the right thumb. (A) Dorsal view. (B) Lateral view.

**Figure 2:**
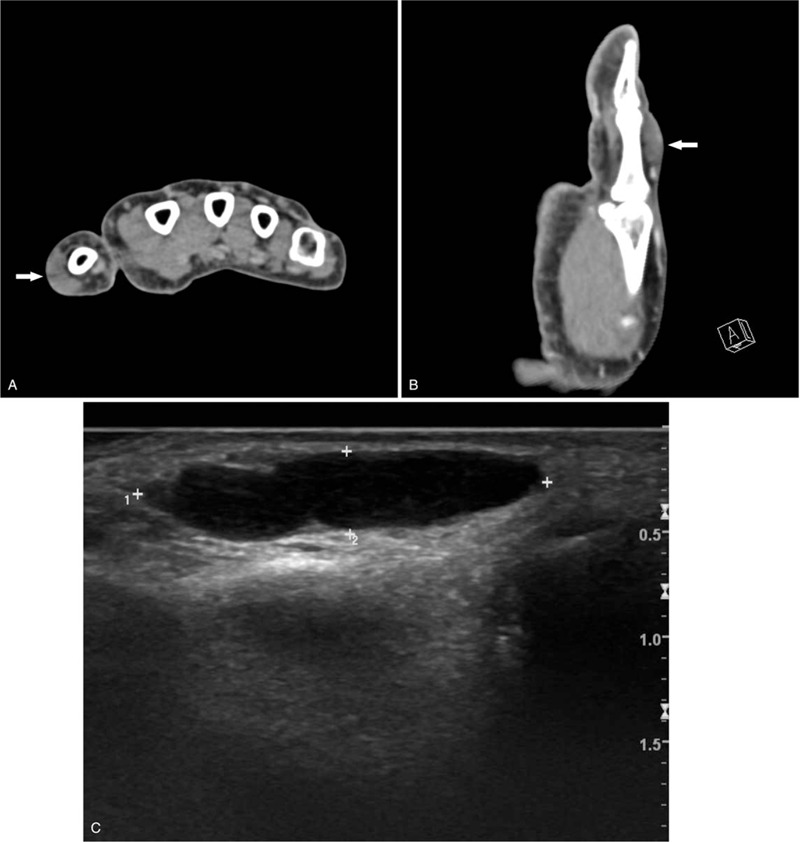
Preoperative CT and US images. (A), (B) Preoperative CT images. (C) Preoperative US image. CT = computed tomography, US = ultrasonography.

A 60-year-old woman presented with a slow-growing mass on the proximal phalanx of the right thumb for over a decade (Fig. 1). The mass was hard, with a non-tender and non-compressible swelling. The patient did not experience pain but complained of numbness in her right thumb. She was undergoing pharmacological treatment for stroke in the right thalamus and for hypertension. Preoperative imaging revealed a moderately defined soft tissue mass, which measured 1.5 cm × 1.5 cm × 0.7 cm, with no bone and joint abnormalities on the right thumb (Fig. 2). This was clinically misdiagnosed as a ganglion or giant cell tumor of the tendon sheath.

**Figure 3:**
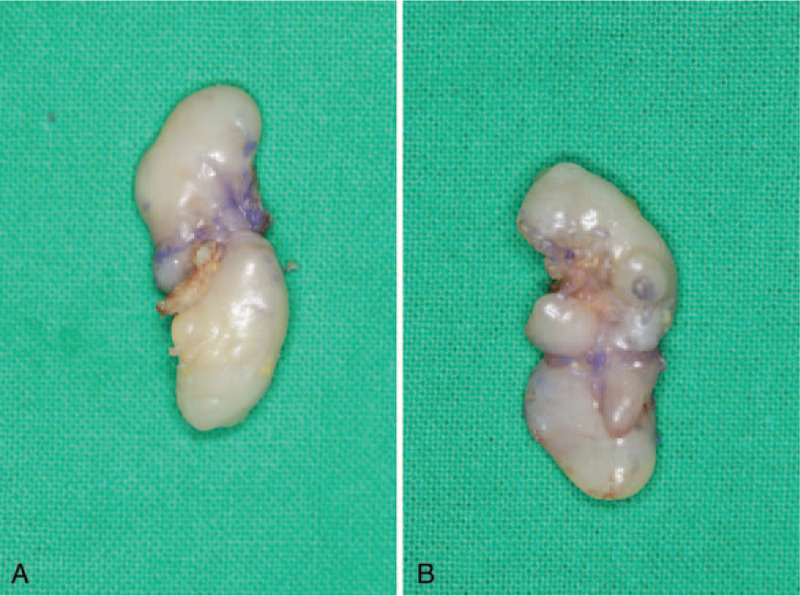
Excised specimen. (A) Dorsal view. (B) Palmar view.

Under general anesthesia, we performed complete surgical excision of the tumor. A longitudinal incision was made through the dorsal side of the proximal phalanx. The tumor appeared to be well-circumscribed and progressed along the dorsal digital branch of the radial nerve of the thumb. The tumor was removed entirely by delicate dissection (Fig. 3).

**Figure 4:**
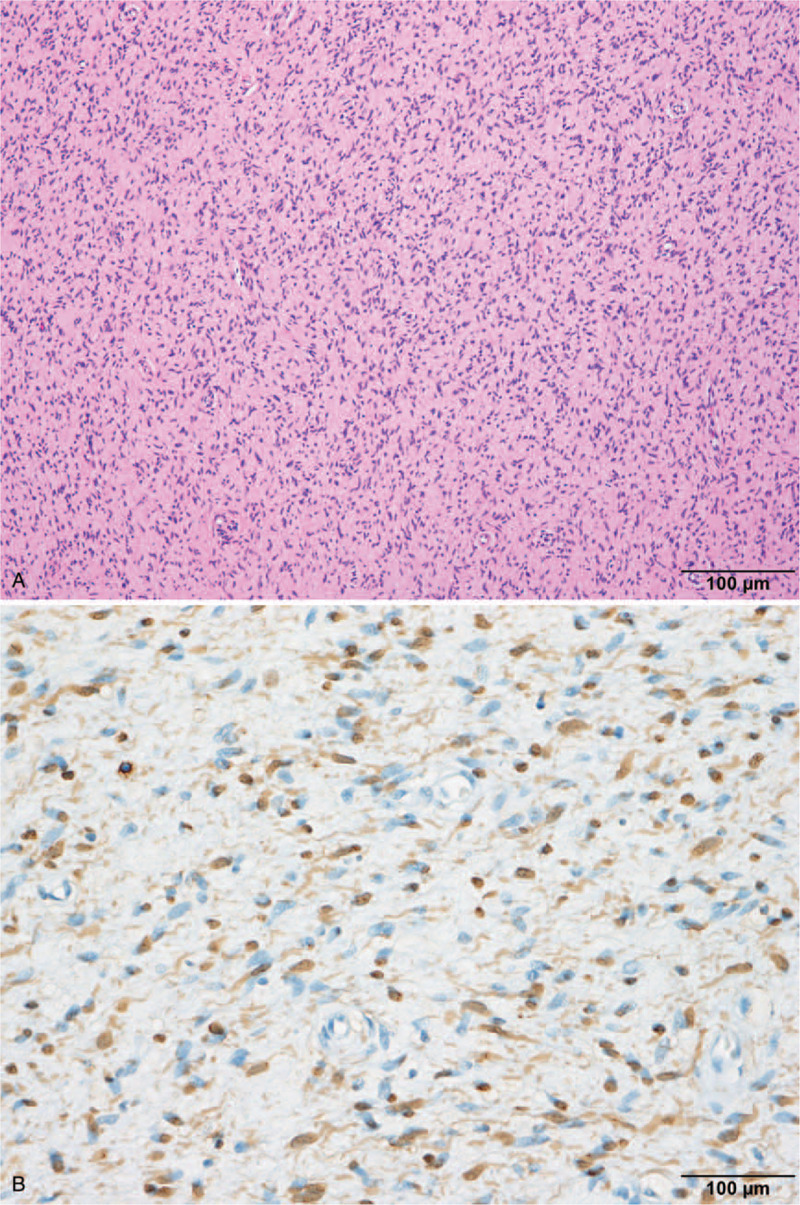
Photomicrographs of the mass. (A) Histopathological examination reveals randomly oriented spindle cells with wavy, hyperchromatic nuclei and thin and thick collagen strands (hematoxylin and eosin stain, 200×). (B) Immunohistochemical examination shows S100 protein staining positive in tumor cells (400×).

Histopathological examination showed randomly oriented spindle cells with wavy, hyperchromatic nuclei and thin and thick collagen strands (Fig. 4A). Immunohistochemically, the tumor expressed S-100 protein and neuron-specific enolase but was not reactive to epithelial membrane antigen (Fig. 4B). A diagnosis of solitary neurofibroma was confirmed, without evidence of neurofibromatosis.

**Figure 5:**
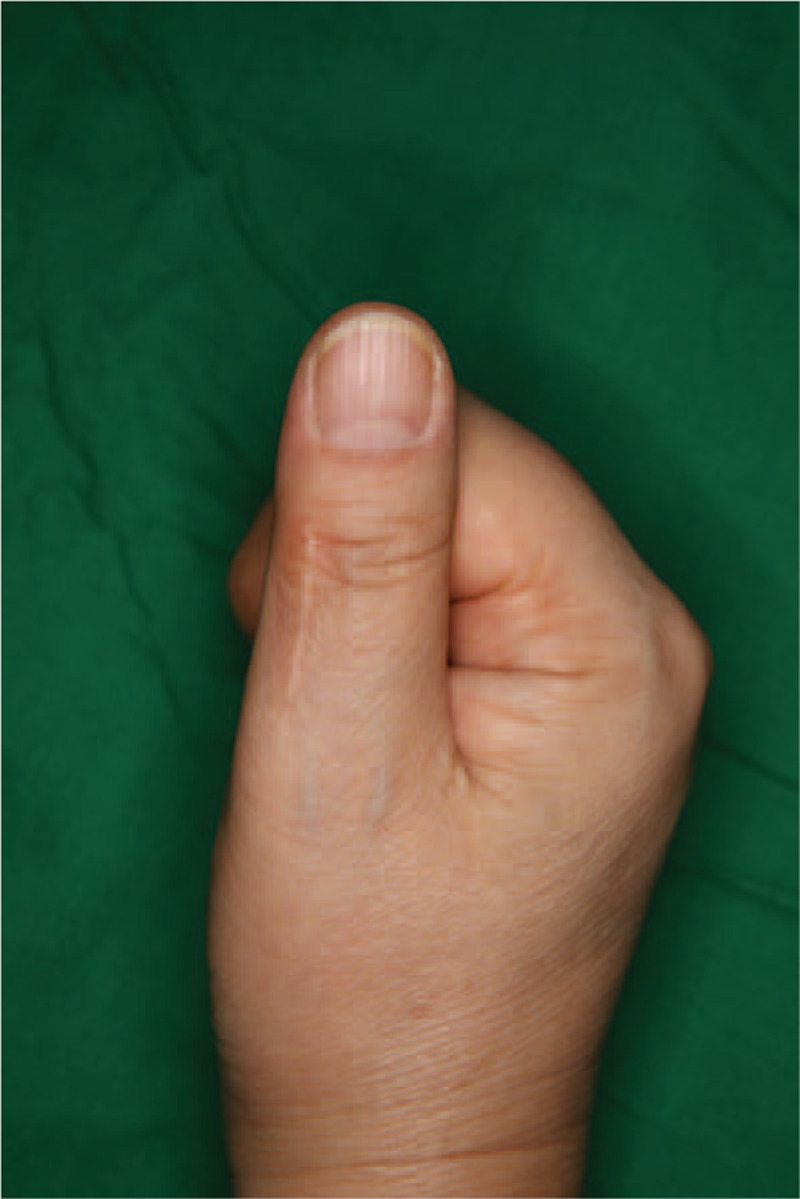
Fourteen-month postoperative view.

After surgery, the patient did not complain of pain but had a temporary tingling sensation. After 14 months of follow-up, no signs of recurrence of the tumor were observed (Fig. 5).

We obtained the patient's medical records and reviewed the related literature. Informed written consent was obtained from the patient for publication of this case report and accompanying images. This study was approved by the Institutional Review Board of Chonnam National University Hospital (CNUH-2019-011) and was conducted in accordance with the principles of the Helsinki Declaration II.

## 3 Discussion

Solitary neurofibromas usually develop from small nerves and show a slow growth pattern, often without obvious symptoms.^[4]^ Lee et al^[3]^ reviewed the medical records of patients diagnosed as having a solitary neurofibroma between January 2005 and June 2010. Only one patient had a disease duration of more than a decade, and the location of the tumor was the right ankle. In addition, only one case developed in the hand. A solitary neurofibroma growing over a decade in the hand, as observed in this case, has not been reported so far.

Because solitary neurofibromas do not cause unusual symptoms that can be distinguished from other tumors such as ganglion cyst, giant cell tumor of the tendon sheath, epidermal inclusion cyst, lipoma, neuroma, fibroma, and glomus tumor, differential diagnosis is necessary. Although radiological examinations such as ultrasonography (US), computed tomography (CT), and magnetic resonance imaging are important modalities for determining the morphology and location of the soft tissue masses, they are likewise limited in terms of offering a specific diagnosis.^[5]^ In this case, the clinical diagnosis based on the results of CT and US, was ganglion cyst or giant cell tumor of the tendon sheath. These results suggest that the tumors may not be differentiated from solitary neurofibromas radiologically and that they occur more frequently than solitary neurofibroma.

Histological examination is indispensable for definitive diagnosis, and immunohistochemical staining is important. On histopathological examination, solitary neurofibromas demonstrate wavy, spindled nuclei, fine collagen fibers, and a myxoid stroma with an abundance of mast cells.^[6]^ However, the histological appearance varies according to the amount of mucin and myxoid tissue present. In immunohistochemistry, S-100 protein, cholinesterase activity, vimentin, and myelin basic protein are positive markers.^[6]^ In the present case, the histological features typical of neurofibromas, and expressions of S-100 protein, vimentin, and myelin basic protein were noted.

Pain, neurological symptoms, increase in size, and/or functional impairment are indications for surgical treatment.^[7]^ However, most benign nerve sheath tumors are asymptomatic. Hence, it is a treatment dilemma for surgeons whether to attempt surgical excision or to manage with close observation. In this case, the patient did not complain of pain; however, she complained of numbness of her right thumb, which is a neurological symptom and led to the decision to perform complete excision.

Solitary neurofibromas, especially those in the hand, are rare. However, while dealing with soft tissue tumors of the hand, particularly with neurologic symptoms, neurofibroma should be included in the differential diagnosis.

## Author contributions

**Conceptualization:** Kwang Seog Kim.

**Data curation:** Dong Gyu Lee, Do Hun Lee.

**Formal analysis:** Jae Ha Hwang, Sam Yong Lee.

**Investigation:** Dong Gyu Lee, Do Hun Lee.

**Methodology:** Jae Ha Hwang, Sam Yong Lee.

**Project administration:** Kwang Seog Kim.

**Writing – original draft:** Kwang Seog Kim, Dong Gyu Lee, Do Hun Lee.

**Writing – review & editing:** Kwang Seog Kim.

## Abbreviations

Abbreviations: CT = computed tomography, US = ultrasonography.
